# Neighborhood Indices, Income, and Cardiovascular-Kidney-Metabolic Syndrome at the Census Tract Level

**DOI:** 10.1001/jamanetworkopen.2026.6019

**Published:** 2026-04-09

**Authors:** Vaishnavi Krishnan, Xiaoning Huang, Cyanna McGowan, Nilay S. Shah, Farah Qureshi, Cynthia S. Minkovitz, Kiarri N. Kershaw, Alexa A. Freedman, Gregory E. Miller, Donald M. Lloyd-Jones, Sadiya S. Khan

**Affiliations:** 1 Section of Preventive Medicine & Epidemiology, Department of Medicine, Boston University Chobanian & Avedisian School of Medicine, Boston, Massachusetts; 2 Division of Cardiology, Department of Medicine, Northwestern University Feinberg School of Medicine, Chicago, Illinois; 3 Department of Preventive Medicine, Northwestern University Feinberg School of Medicine, Chicago, Illinois; 4 Department of Population, Family and Reproductive Health, Johns Hopkins Bloomberg School of Public Health, Baltimore, Maryland; 5 Institute for Policy Research, Northwestern University, Evanston, Illinois; 6 Department of Psychology, Northwestern University, Evanston, Illinois

## Abstract

**Importance:**

Several integrative indices at the neighborhood level have been developed in the US, but direct comparisons across these indices for the prevalence of cardiovascular-kidney-metabolic (CKM) conditions are limited. Moreover, it is not known whether certain indices better capture place-based variability in CKM conditions or provide additional information when compared with a single measure of income.

**Objective:**

To determine how much variability is explained by neighborhood indices in the prevalence of CKM conditions at the census tract level and the incremental value of each index when added to median household income.

**Design, Setting, and Participants:**

This cross-sectional study of US national surveillance data (Behavioral Risk Factor Surveillance System and American Community Survey) included all census tracts with complete data for exposures, covariates, and outcomes from 2010 to 2022. Analyses were completed between October 2024 and July 2025.

**Exposures:**

Seven neighborhood indices available at the census tract level (Area Deprivation Index, Child Opportunity Index, Environmental Justice Index, Neighborhood Deprivation Index, Social Deprivation Index, Social Vulnerability Index, Structural Racism Effect Index) and median household income at the census tract level from the American Community Survey.

**Main Outcomes and Measures:**

The primary outcome was prevalence of CKM conditions (coronary heart disease [CHD], stroke, and chronic kidney disease [CKD]) at the census tract level. Differences in the exposures and outcomes were visualized by mapping index scores, median household income, and prevalences of CKM conditions for all census tracts. To assess index agreement, pairwise correlations were computed with Spearman rank correlation coefficients, and to assess the incremental variability explained by each index when added to median household income, change in *r*^2^ was calculated.

**Results:**

Of the 65 476 US census tracts included, median (IQR) prevalence was 5.9% (4.7%-7.4%) for CHD, 3.3% (2.6%-4.1%) for stroke, and 2.9% (2.5%-3.4%) for CKD. The indices and income were modestly to highly correlated (range, 0.46-0.94), with some discordance in how each measure classified census tracts by quartiles of index scores. All indices and income were significantly associated with CKM condition prevalence at the census tract level in multivariable linear regression models, adjusted for median population size and age. The *r*^2^ values of the indices with CHD, stroke, and CKD ranged from 0.379 (SE = 0.033) for the correlation between the Environmental Justice Index and stroke to 0.688 (SE = 0.002) for the correlation between the Structural Racism Effect Index and stroke. Additionally, the improvement in variability for CHD, stroke, and CKD (change in *r*^2^) explained by the addition of each index to income ranged from 0.014 (SE = 0.001) for the correlation between the Environmental Justice Index and CHD to 0.195 (SE = 0.002) for the correlation between Structural Racism Effect Index and stroke.

**Conclusions and Relevance:**

In this cross-sectional study, there were similar associations across neighborhood indices and income with the prevalence of CKM conditions. These findings inform how different place-based measures can be applied in public health research and policy.

## Key Points

**Question:**

How much variability is explained by neighborhood indices and median household income for the prevalence of cardiovascular-kidney-metabolic (CKM) conditions at the census tract level?

**Findings:**

In this cross-sectional study of 65 476 census tracts, the 7 neighborhood indices examined and median household income were all modestly to highly correlated. While there was moderate discordance when classifying census tracts by quartiles of index scores, all had similar magnitudes of association with the prevalence of CKM conditions, including coronary heart disease, stroke, and chronic kidney disease.

**Meaning:**

These findings suggest that identifying advantages and disadvantages of each neighborhood measure can inform utility and selection of specific place-based measures for research and policy.

## Teaser

This cross-sectional study estimates the association of 7 different neighborhood indices with the prevalence of cardiovascular-kidney-metabolic syndrome at the census tract level.

## Introduction

Cardiovascular-kidney-metabolic (CKM) syndrome is a health disorder due to interrelated conditions, which include obesity, diabetes, hypertension, kidney disease, and cardiovascular diseases. These CKM conditions confer a substantial public health burden, with significant morbidity, mortality, and health care costs.^1,2^ Clinical risk factors for CKM conditions at the individual level are well established, and, in recent years, strong evidence has emerged for nonclinical or social determinants of health.^3,4,5^ Among these, the influence of social, environmental, and economic characteristics of the neighborhoods in which people live on CKM conditions has garnered particular interest. To capture these multiple domains, integrative indices have been developed and aim to provide a holistic assessment of the neighborhood environment and its influence on health beyond a single measure of socioeconomic status (eg, household income).^6^ Use of neighborhood indices has also been embedded in the primary prevention of cardiovascular diseases with the inclusion of the Social Deprivation Index (SDI) in the novel Predicting Risk of Cardiovascular Disease Events equations.^7^

Many neighborhood indices exist that have been developed with varying methodologies and for different purposes, yet many include similar measures and share a common goal of measuring place-based context. Consequently, it is of growing interest to understand whether specific indices have incremental value when compared with a simpler measure of neighborhood social context, the most commonly used of which is median household income. Moreover, while several indices have been individually studied across several of the CKM conditions (including the Area Deprivation Index [ADI],^8,9^ Child Opportunity Index [COI],^10^ Environmental Justice Index [EJI],^11^ Neighborhood Deprivation Index [NDI],^12^ SDI,^13^ Structural Racism Effect Index [SREI],^14^ and Social Vulnerability Index [SVI]^15,16^), direct comparison of these indices has not been explored for CKM conditions.

Therefore, our objectives were to examine (1) the correlation and agreement across 7 neighborhood indices and median household income at the census tract level, (2) the associations of each index and median household income with the prevalence of several CKM conditions at the census tract level, and (3) whether 1 or more indices better capture census tract–level variability in each CKM condition compared with median household income.

## Methods

This cross-sectional study used aggregated publicly available data at the census tract level and was deemed exempt from review and the requirement of informed consent by the institutional review board at Northwestern University Feinberg School of Medicine. All methods followed the Strengthening the Reporting of Observational Studies in Epidemiology (STROBE) reporting guideline.

### Data Sources

Seven neighborhood indices that are available or can be calculated at the census tract level were identified: ADI (2015 version),^17,18,19^ COI 3.0 (2019 version),^20,21^ EJI (2022 version),^22^ NDI (2013-2017 version),^23^ SDI (2019 version),^24^ SREI (2023 version),^14,25^ and SVI (2018 version).^26^ Some of these indices have been commonly used in health outcomes research (ADI, COI, NDI, SDI, and SVI) while others are novel (SREI and EJI). All census tract–level data to calculate the indices are publicly available for download. eTable 1 in Supplement 1 summarizes the purpose of each index and the included domains, component variables, index scoring, data sources, and years of data. All indices include variables for place-based measures of educational attainment, employment, poverty, housing, and household characteristics, with additional variables included in COI, EJI, SREI, and SVI (eTable 2 in Supplement 1).

Median household income and covariates (median age, total population size) for each census tract were obtained from 2015 to 2019 American Community Survey data.^27^ Crude prevalence rates of CKM conditions at the census tract level were obtained from the 2021 Centers for Disease Control Population Level Analysis and Community Estimates dataset, and data are publicly available for download.^28^ The primary outcomes in our analysis included coronary heart disease (CHD), stroke, and chronic kidney disease. Secondary outcomes included obesity, hypertension, and diabetes. These measures were derived from self-reported data collected in the 2019 Behavioral Risk Factor Surveillance System and the 2015 to 2019 American Community Survey.^28^

### Statistical Analysis

The unit of measurement for exposures in this analysis was the census tract level. Therefore, all analyses were conducted at the census tract level because a census tract is a small geographic area that was defined by the US Census Bureau to represent a neighborhood or area with shared characteristics. Census tracts with missing data (for exposures, covariates, or outcomes) were excluded in the final sample (6861 of the 72 337 census tracts available in the Centers for Disease Control Population Level Analysis and Community Estimates) (eTable 3 in Supplement 1). Of the included indices, the ADI provides data for census block groups, so scores were aggregated to the census tract level for comparability using population-weighted means as previously described.^29^ For the EJI, the Social-Environmental Ranking score of the EJI was used in our analyses as recommended in the methodological documentation when evaluating health outcomes.^22^ To ensure consistent comparisons, with higher scores representing higher degrees of deprivation across all measures in our analyses, the COI and income were inversely analyzed.

First, differences in the exposures and outcomes were visualized by mapping index scores, median household income, and prevalences of CKM conditions for all census tracts included in our analysis using 2019 TIGER/Line (Master Address File/Topologically Integrated Geographic Encoding and Referencing) shapefiles for census tracts from the US Census Bureau.^30^ Maps were scaled by deciles of exposures and outcomes to facilitate comparison given differences in scoring. To assess index agreement, pairwise correlations between crude index scores and median household income were computed with Spearman rank correlation coefficients. Additionally, because census tracts and index scores are often described by quantiles in research and policy, we examined the proportion of census tracts that would be classified within the same quartile by indices or median household income (ie, quartile agreement).

Next, all exposures and outcomes were standardized to examine the associations of indices and median household income with the prevalence of each CKM condition at the census tract level using linear regression models, adjusted for total population and median age. To assess the incremental variability explained by each index when added to median household income, change in *r*^2^ was calculated after each index was added as a covariate to the adjusted regression model evaluating median household income as the primary independent variable with the CKM condition prevalence as the dependent variable. Because all indices include socioeconomic variables, potential collinearity in these models was evaluated using variance inflation factors, with values of 10 or greater considered indicative of collinearity.^31^ Secondary analyses were conducted to examine which subdomains contributed most significantly to explaining census tract–level variability for the top performing index based on *r*^2^ and change in *r*^2^ values. In adjusted models, the associations of subdomains with the prevalence of each CKM condition were examined and change in *r*^2^ was calculated after each subdomain was added to a base model with the remaining subdomains. Potential collinearity in these models was also assessed with variance inflation factors.

All statistical analyses were performed on R statistical software version 4.2.3 (R Project for Statistical Computing), and 2-sided *P* values less than .05 were considered statistically significant. Analyses were completed between October 2024 and July 2025.

## Results

### Census Tract–Level Characteristics

**Figure 1.**
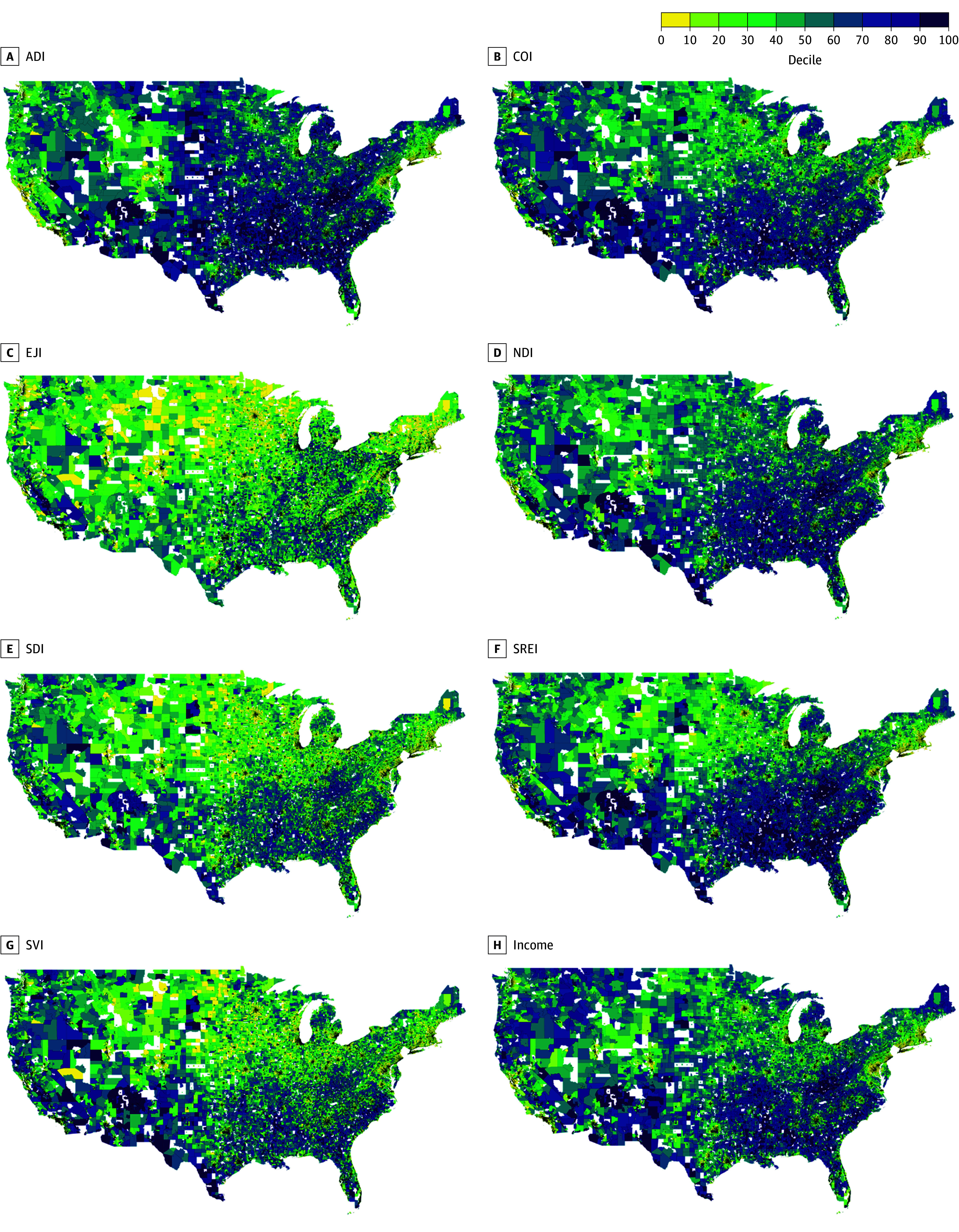
Maps of Geographic Heterogeneity in 7 Neighborhood Indices and Median Household Income at the Census Tract Level Maps of census tract–level data for Area Deprivation Index (ADI; A); Child Opportunity Index (COI; B); Environmental Justice Index (EJI; C); Neighborhood Deprivation Index (NDI; D); Social Deprivation Index (SDI; E); Structural Racism Effect Index (SREI; F); Social Vulnerability Index (SVI; G); and median household income (H). The color scale represents deciles of crude, unstandardized index scores and median household income. Darker shading (ie, higher deciles) represents higher levels of deprivation.

Among 65 476 census tracts included in our analysis, median (IQR) population size was 4203 (3015-5632) individuals, age was 39.3 (34.5-44.3) years, and household income was $58 870 ($44 276-$79 802) across census tracts (Table 1). The median (IQR) prevalence of the primary CKM outcomes was 5.9% (4.7%-7.4%) for CHD, 3.3% (2.6%-4.1%) for stroke, and 2.9% (2.5%-3.4%) for chronic kidney disease. There was geographic heterogeneity in the index scores and median household income across the US as visualized in Figure 1 and for the prevalence of CKM conditions as visualized in eFigure 1 in Supplement 1.

**Table 1.** Characteristics of US Census Tracts^a^

Characteristic	Estimate, median (IQR)
Total population	4203 (3015 to 5632)
Median age, y	39.3 (34.5 to 44.3)
Median household income, $	58 870 (44 276 to 79 802)
Index scores	
Area Deprivation Index	52.00 (28.44 to 73.87)
Child Opportunity Index	45.00 (22.00 to 70.00)
Environmental Justice Index	0.50 (0.25 to 0.75)
Neighborhood Deprivation Index	0.13 (−0.65 to 0.76)
Social Deprivation Index	54.0 (29.0 to 78.0)
Structural Racism Effect Index	0.07 (−0.57 to 0.79)
Social Vulnerability Index	0.51 (0.26 to 0.75)
Cardiovascular-kidney-metabolic conditions prevalence, %	
Coronary heart disease	5.9 (4.7 to 7.4)
Stroke	3.3 (2.6 to 4.1)
Chronic kidney disease	2.9 (2.5 to 3.4)
Obesity	32.9 (28.3 to 37.1)
Hypertension	32.0 (27.8 to 36.8)
Diabetes	10.4 (8.6 to 12.8)

^a^ After excluding for missing exposures, covariates, or outcomes, 65 476 census tracts were included in the final sample. Index scores represent crude, unstandardized values. Data on total population, median age, and median household income were obtained from the 2015 to 2019 American Community Survey. Prevalence data for cardiovascular-kidney-metabolic conditions were obtained from the Centers for Disease Control and Prevention Population Level Analysis and Community Estimates (2019 Behavioral Risk Factor Surveillance System and 2015-2019 American Community Survey).

### Correlation and Agreement Across Census Tract–Level Measures

**Figure 2.**
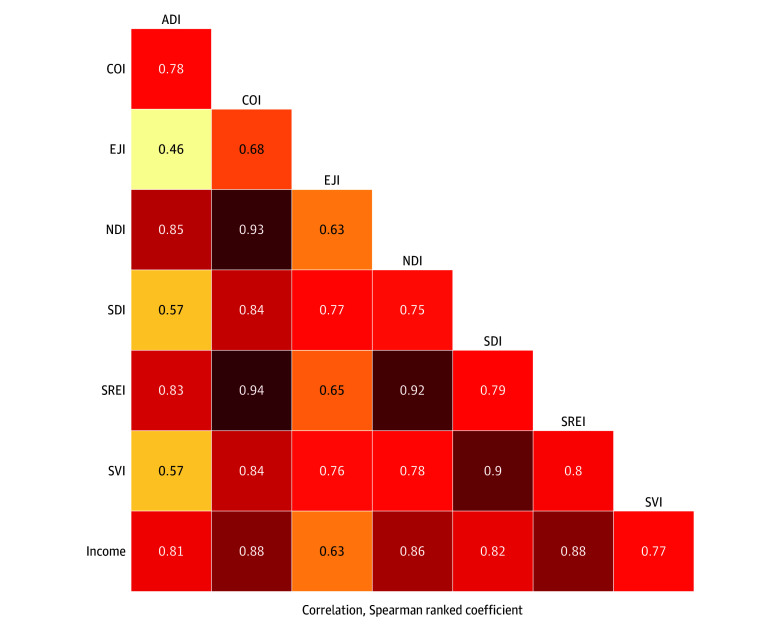
Heatmap of Correlations of Neighborhood Indices and Median Household Income at the Census Tract Level Heatmap for pairwise analyses across crude index scores and median household income at the census tract level using Spearman ranked correlation coefficients. Darker shading represents higher values for correlation. ADI indicates Area Deprivation Index; COI, Child Opportunity Index; EJI, Environmental Justice Index; NDI, Neighborhood Deprivation Index; SDI, Social Deprivation Index; SREI, Structural Racism Effect Index; SVI, Social Vulnerability Index.

Pairwise correlations for neighborhood index scores and median household income across census tracts are presented in Figure 2 and ranged from 0.46 (ADI vs EJI) to 0.94 (SREI vs COI). Additionally, correlation of each index with median household income ranged from 0.63 (EJI) to 0.88 (COI and SREI). The proportion of census tracts that would be classified within the same quartile by 2 different indices or income is presented in eFigure 2 in Supplement 1. Overall, pairwise quartile agreement of index scores and median household income ranged from 38.4% for the ADI and EJI (ie, 38.4% of census tracts would be classified as being in the same quartile of neighborhood deprivation when measuring using the ADI vs EJI) to 74.1% for the COI and SREI.

### Associations With CKM Conditions

The distribution of the neighborhood indices and income after standardization is shown in eFigure 3 in Supplement 1. All indices and median household income were significantly associated with the census tract–level prevalence of the primary CKM outcomes in adjusted models. The magnitude of the correlation (*r*^2^) between each index and the prevalence of the CKM outcomes varied from 0.379 (SE = 0.003) for the correlation between the EJI and stroke to 0.688 (SE = 0.002) for the correlation between the SREI and stroke (Table 2). For example, the *r*^2^ (SE) values between each neighborhood index and CHD ranged from 0.390 (0.003) for the EJI to 0.675 (0.002) for the SREI, compared with 0.586 (0.002) for median household income.

**Table 2.** Neighborhood Indices, Median Household Income, and Cardiovascular-Kidney-Metabolic Outcomes at the Census Tract Level^a^

Index	Coronary heart disease		Stroke		Chronic kidney disease	
	β (SE)	*r*^2^ (SE)	β (SE)	*r*^2^ (SE)	β (SE)	*r*^2^ (SE)
Area Deprivation Index	0.687 (0.002)	0.636 (0.002)	0.665 (0.003)	0.519 (0.003)	0.634 (0.003)	0.469 (0.003)
Child Opportunity Index	0.714 (0.003)	0.637 (0.002)	0.767 (0.003)	0.612 (0.002)	0.793 (0.003)	0.633 (0.002)
Environmental Justice Index	0.468 (0.003)	0.390 (0.003)	0.563 (0.003)	0.379 (0.003)	0.601 (0.003)	0.402 (0.003)
Neighborhood Deprivation Index	0.702 (0.003)	0.638 (0.002)	0.728 (0.003)	0.579 (0.002)	0.735 (0.003)	0.574 (0.002)
Social Deprivation Index	0.670 (0.003)	0.530 (0.003)	0.744 (0.003)	0.512 (0.003)	0.799 (0.003)	0.561 (0.002)
Structural Racism Effect Index	0.739 (0.002)	0.675 (0.002)	0.817 (0.002)	0.688 (0.002)	0.814 (0.002)	0.671 (0.002)
Social Vulnerability Index	0.633 (0.003)	0.544 (0.003)	0.709 (0.003)	0.537 (0.003)	0.768 (0.003)	0.597 (0.002)
Median household income	0.653 (0.003)	0.586 (0.002)	0.652 (0.003)	0.493 (0.002)	0.663 (0.003)	0.492 (0.002)

^a^ Values represent β coefficients and *r*^2^ values from multivariable linear regression models between neighborhood measures and cardiovascular-kidney-metabolic outcomes, adjusted for census tract population and median age. Bootstrapping with 500 replicates was conducted for the SE for *r*^2^ values. All neighborhood measures and cardiovascular-kidney-metabolic outcomes were standardized before linear regression was performed. All neighborhood measures were significantly associated with cardiovascular-kidney-metabolic outcomes (*P* < .05).

The change in *r*^2^ after the addition of each index individually to the adjusted models with median household income varied for the prevalence of primary outcomes from 0.014 (SE = 0.001) for the correlation between the EJI and CHD to 0.195 (SE = 0.002) for the correlation between the SREI and stroke (Table 3). For CHD, change in *r*^2^ values (SE) ranged from 0.014 (0.001) for the EJI to 0.100 (0.002) for the SREI. Similar patterns were observed for secondary outcomes of CKM risk factors (obesity, hypertension, diabetes) (eTables 4-5 in Supplement 1) and in unadjusted models (eTable 6 in Supplement 1).

**Table 3.** Change in *r*^2^ Values With Addition of Neighborhood Indices to Median Household Income for Cardiovascular-Kidney-Metabolic Outcomes at the Census Tract Level^a^

Index	Change in *r*^2^ (SE)		
	Coronary heart disease	Stroke	Chronic kidney disease
Area Deprivation Index	0.086 (0.002)	0.070 (0.002)	0.044 (0.001)
Child Opportunity Index	0.071 (0.001)	0.124 (0.002)	0.144 (0.002)
Environmental Justice Index	0.014 (0.001)	0.049 (0.001)	0.065 (0.001)
Neighborhood Deprivation Index	0.068 (0.001)	0.094 (0.002)	0.092 (0.002)
Social Deprivation Index	0.027 (0.001)	0.067 (0.001)	0.102 (0.002)
Structural Racism Effect Index	0.100 (0.002)	0.195 (0.002)	0.179 (0.002)
Social Vulnerability Index	0.046 (0.001)	0.097 (0.001)	0.142 (0.002)

^a^ Changes in *r*^2^ values were derived after each neighborhood index was individually added to multivariable linear regression models between median household income and cardiovascular-kidney-metabolic outcomes, adjusted for census tract population and median age. All variance inflation factors for these models (median household income + index + covariates) were less than 5. Bootstrapping with 500 replicates was conducted for SEs. All neighborhood measures and cardiovascular-kidney-metabolic outcomes are estimated at the census tract level and were standardized before linear regression was performed.

### Analyzing Subdomains for the SREI

For all CKM conditions analyzed, *r*^2^ and change in *r*^2^ values were highest for the SREI; therefore, the subdomains of the SREI were further explored to determine which contributed most significantly to these findings. All SREI subdomains were significantly associated with CKM conditions in adjusted models, with *r*^2^ values ranging from 0.167 to 0.664 for the primary outcomes (eTable 7 in Supplement 1). The SREI subdomain with the highest *r*^2^ values for CHD, stroke, and chronic kidney disease was income and poverty. eTable 8 in Supplement 1 presents the change in *r*^2^ values after the built environment, criminal justice, social cohesion, and transportation subdomains were each added to a base model with the rest of the subdomains that focused on socioeconomic variables (education, employment, housing, income and poverty, and wealth). In the base model, the *r*^2^ (SE) values for the primary CKM outcomes ranged from 0.655 (0.002) for stroke to 0.690 (0.002) for CKD, with minimal increases in *r*^2^ when additional subdomains were added (change in *r*^2^ <0.1).

## Discussion

In this nationwide cross-sectional study of neighborhood measures and CKM conditions at the census tract level, we demonstrated 4 key findings. First, each of the 7 indices were highly correlated with each other and with a single measure of socioeconomic status, median household income. Second, the magnitude of the associations of neighborhood measures (indices and income) with the prevalence of each CKM condition at the census tract level was similar. Third, despite this strong correlation, modest discordance was noted in classification of census tracts within the same quartile of deprivation across measures. Fourth, a large proportion of the place-based variability in the prevalence of each CKM outcome was captured by median household income alone. However, some indices had minimal to modest significant improvement in the change in *r*^2^ when added to income.

The current study builds on prior work that has explored the association of place-based factors with a variety of non-CKM outcomes, including for COVID-19, malignant neoplasms, and pediatric health.^29,32,33,34,35,36,37,38,39,40^ We newly focused on CKM conditions and found that the neighborhood indices included in our study had largely similar magnitudes of association with CKM condition prevalence that were also similar to median household income. Moreover, our analysis of index subdomains affirmed that the majority of census tract–level variability explained by the SREI (the index with the highest *r*^2^ values) appears to arise from domains representing socioeconomic variables that are commonly included in neighborhood measures of deprivation (individually or in an index). This finding is consistent with prior research demonstrating that poverty, educational attainment, and employment have an outsized impact on health outcomes, and that these place-based variables contribute most significantly to the performance of overall index scores^3,21,32^; this suggests that future research should prioritize federal, state, and local policies that address these key domains.

Although additional domains may play an important role in the development of adverse health outcomes, a greater number of variables in an index or score may not inherently improve its utility. Among the indices we studied, the total number of variables (ranging from 7 to 44 variables) did not consistently predict index performance, and a simple measure of median household income performed similarly to neighborhood indices. Median household income at the neighborhood level is frequently included in indices and also influences other index subdomains (eg, educational attainment, employment, and housing status).^3^ Given that median household income is often easier to access and interpret compared with composite indices, median household income may be the preferred and simplest choice for many objectives. However, analysis of income alone may not be able to inform policy-level interventions that aim to improve social and environmental factors in neighborhoods and move beyond solutions that rely solely on economic investment or cash transfers. On the other hand, although indices may offer comprehensive approaches to capture neighborhood social context beyond income, the associations of individual index domains with health outcomes are already well established, and a greater gap exists in understanding how these place-based findings can be best translated into sustainable and impactful interventions across multiple domains.^3^

Therefore, assessing the goals of applying neighborhood measures in research and public policy are crucial when considering which measures are included and implemented, and should be a key focus of future research. Moreover, which variables are included in an index should be considered in the context of potential harms because not all variables are modifiable and cannot be considered as factors that may be intervened upon. For example, caution must be exercised when interpreting indices that include demographic variables such as age, race and ethnicity, and language status.^41^ Even if they improve index performance and demonstrate associations with outcomes, such sociodemographic variables serve as proxies for underlying social and structural barriers that drive neighborhood-level disparities. Their inclusion has the potential for misinterpretation by overlooking what they seek to represent or may infer an immutable biological basis for observed differences.^41^ Consequently, modifiable place-based factors that represent actionable policy levers (ie, community infrastructure and resources) should be prioritized in index design to avoid unintentional harm.

Finally, how these findings are interpreted and guide subsequent policy decisions depends on the intended goal. For example, the ADI and SVI were both used to inform equitable vaccine allocation during the COVID-19 pandemic and demonstrated similar associations across outcomes, but may prioritize different neighborhoods due to varying index purposes and variable weighting.^29,33,40,42^ In our analysis, over one-half of census tracts would be classified as different quartiles by the ADI vs SVI, despite comparable performance overall at the population level. These findings underscore the importance of informed index selection, especially at greater levels of granularity when differences in how neighborhood deprivation is quantified can impact which communities receive a particular intervention. In observational research, aligning the measures and the question being studied (ie, educational resources, structural racism, and environmental health) can inform the a priori selection of the index to be used. In particular, theory-driven research can identify constructs distinct from traditional socioeconomic status indicators that may be uniquely studied to identify pathways to target for policy-level interventions.^43^

### Limitations

There are some limitations to note. First, there were some small differences in temporal harmonization for each index based on data availability. However, we prioritized alignment of data sources with the same decennial census tract boundaries and derived from pre–COVID-19 pandemic data. Second, the CKM conditions included are modeled based on self-report, which may lead to underestimation. Moreover, there may be differential rates of underestimation in areas with higher deprivation, but this would bias findings toward the null. Third, covariates of age and population size in our analyses may be accounted for in some of the neighborhood indices, which may lead to overadjustment, but similar patterns were observed in unadjusted analyses. Fourth, we conducted a complete case analysis and excluded approximately 9.5% of census tracts from the analysis for missing data on exposures. However, more than 60 000 census tracts were available for the analysis representing adequate power with broad continental US geographic representation. Fifth, this study only included census tract–level factors. Additional variability may be captured by individual-level social factors.

## Conclusions

In this cross-sectional study, a diverse set of 7 neighborhood indices and median household income were highly correlated and similarly associated with the prevalence of CKM conditions at the census tract level. However, each measure resulted in modest differential classification of neighborhoods within the same quartile of deprivation. Future research should focus on clarifying the goals when incorporating place-based measures to align index selection for research and policy.

## Notes

Supplement 1. eTable 1. Domains, variables, and methodology for each neighborhood index eTable 2. Comparison of selected domains included in seven neighborhood indices eTable 3. Characteristics of US census tracts excluded in analysis eFigure 1. Geographic heterogeneity in cardiovascular-kidney-metabolic conditions at the census tract-level eFigure 2. Quartile agreement between neighborhood indices and median household income at the census tract-level eFigure 3. Distribution of standardized neighborhood index scores and median household income at the census tract-level across the US eTable 4. Associations between neighborhood indices, median household income, and cardiovascular-kidney-metabolic risk factors at the census tract-level eTable 5. Change in R2 values with addition of neighborhood indices to median household income for cardiovascular-kidney-metabolic risk factors at the census tract-level eTable 6. Unadjusted associations between neighborhood indices, median household income, and cardiovascular-kidney-metabolic conditions at the census tract-level eTable 7. Associations between SREI subdomains and cardiovascular-kidney-metabolic conditions at the census tract-level eTable 8. Change in R2 values for Structural Racism Effect Index subdomains across cardiovascular-kidney-metabolic conditions at the census tract-level eReferences

Supplement 2. Data Sharing Statement

## References

[1] Ndumele CE, Rangaswami J, Chow SL, ; American Heart Association. Cardiovascular-kidney-metabolic health: a presidential advisory from the American Heart Association. Circulation. 2023;148(20):1606-1635. doi:10.1161/CIR.0000000000001184 37807924

[2] Ndumele CE, Neeland IJ, Tuttle KR, ; American Heart Association. A synopsis of the evidence for the science and clinical management of cardiovascular-kidney-metabolic (CKM) syndrome: a scientific statement From the American Heart Association. Circulation. 2023;148(20):1636-1664. doi:10.1161/CIR.0000000000001186 37807920

[3] Kershaw KN, Magnani JW, Diez Roux AV, ; Council on Quality of Care and Outcomes Research; Council on Epidemiology and Prevention; Council on Clinical Cardiology; Council on Hypertension; Council on Cardiovascular and Stroke Nursing; Council on Peripheral Vascular Disease; and Council on the Kidney in Cardiovascular Disease. Neighborhoods and cardiovascular health: a scientific statement from the American Heart Association. Circ Cardiovasc Qual Outcomes. 2024;17(1):e000124. doi:10.1161/HCQ.0000000000000124 38073532

[4] Zhu R, Wang R, He J, . Prevalence of cardiovascular-kidney-metabolic syndrome stages by social determinants of health. JAMA Netw Open. 2024;7(11):e2445309. doi:10.1001/jamanetworkopen.2024.45309 39556396 PMC11574692

[5] Li J, Lei L, Wang W, . Social risk profile and cardiovascular-kidney-metabolic syndrome in US adults. J Am Heart Assoc. 2024;13(16):e034996. doi:10.1161/JAHA.124.034996 39136302 PMC11963957

[6] Trinidad S, Brokamp C, Mor Huertas A, . Use of area-based socioeconomic deprivation indices: a scoping review and qualitative analysis. Health Aff (Millwood). 2022;41(12):1804-1811. doi:10.1377/hlthaff.2022.00482 36469826

[7] Khan SS, Matsushita K, Sang Y, ; Chronic Kidney Disease Prognosis Consortium and the American Heart Association Cardiovascular-Kidney-Metabolic Science Advisory Group. Development and validation of the American Heart Association’s PREVENT equations. Circulation. 2024;149(6):430-449. doi:10.1161/CIRCULATIONAHA.123.067626 37947085 PMC10910659

[8] Berman AN, Biery DW, Ginder C, . Association of socioeconomic disadvantage with long-term mortality after myocardial infarction: the Mass General Brigham YOUNG-MI registry. JAMA Cardiol. 2021;6(8):880-888. doi:10.1001/jamacardio.2021.0487 34009238 PMC8135064

[9] Kurani SS, Heien HC, Sangaralingham LR, . Association of area-level socioeconomic deprivation with hypoglycemic and hyperglycemic crises in US adults with diabetes. JAMA Netw Open. 2022;5(1):e2143597. doi:10.1001/jamanetworkopen.2021.43597 35040969 PMC8767428

[10] Aris IM, Rifas-Shiman SL, Jimenez MP, . Neighborhood child opportunity index and adolescent cardiometabolic risk. Pediatrics. 2021;147(2):e2020018903. doi:10.1542/peds.2020-018903 33479165 PMC7906069

[11] Khadke S, Kumar A, Al-Kindi S, . Association of environmental injustice and cardiovascular diseases and risk factors in the United States. J Am Heart Assoc. 2024;13(7):e033428. doi:10.1161/JAHA.123.033428 38533798 PMC11179791

[12] Laraia BA, Karter AJ, Warton EM, Schillinger D, Moffet HH, Adler N. Place matters: neighborhood deprivation and cardiometabolic risk factors in the Diabetes Study of Northern California (DISTANCE). Soc Sci Med. 2012;74(7):1082-1090. doi:10.1016/j.socscimed.2011.11.036 22373821 PMC3437771

[13] Cotton A, Salerno PRVO, Deo SV, . The association between county-level premature cardiovascular mortality related to cardio-kidney-metabolic disease and the social determinants of health in the US. Sci Rep. 2024;14(1):24984. doi:10.1038/s41598-024-73974-9 39443546 PMC11500108

[14] Dyer Z, Alcusky MJ, Galea S, Ash A. Measuring the enduring imprint of structural racism on American neighborhoods. Health Aff (Millwood). 2023;42(10):1374-1382. doi:10.1377/hlthaff.2023.00659 37782878 PMC10804769

[15] Khan SU, Javed Z, Lone AN, . Social vulnerability and premature cardiovascular mortality among US counties, 2014 to 2018. Circulation. 2021;144(16):1272-1279. doi:10.1161/CIRCULATIONAHA.121.054516 34662161

[16] Brush JE Jr, Kim C, Liu Y, . Association between neighborhood-level social vulnerability and hypertension outcomes. JACC Adv. 2025;4(8):101912. doi:10.1016/j.jacadv.2025.101912 40627892 PMC12272890

[17] University of Wisconsin School of Medicine and Public Health. 2015 Area Deprivation Index version 3.1. Accessed January 28, 2025. https://www.neighborhoodatlas.medicine.wisc.edu/

[18] Kind AJH, Buckingham WR. Making neighborhood-disadvantage metrics accessible—the neighborhood atlas. N Engl J Med. 2018;378(26):2456-2458. doi:10.1056/NEJMp1802313 29949490 PMC6051533

[19] Kind AJ, Jencks S, Brock J, . Neighborhood socioeconomic disadvantage and 30-day rehospitalization: a retrospective cohort study. Ann Intern Med. 2014;161(11):765-774. doi:10.7326/M13-2946 25437404 PMC4251560

[20] Child opportunity index 3.0-2021 census tract data for 2010 census tracts. Diversitydatakids.org. Accessed January 28, 2025. https://www.diversitydatakids.org/research-library/child-opportunity-index-30-2021-census-tract-data

[21] Noelke C, McArdle N, DeVoe B, . Child opportunity index 3.0 technical documentation. Diversitydatakids.org. Accessed February 25, 2026. https://diversitydatakids.org/research-library/coi-30-technical-documentation.

[22] Agency for Toxic Substances Disease Registry. Environmental Justice Index data download. Accessed January 28, 2025. https://atsdr.cdc.gov/place-health/php/eji/eji-data-download.html

[23] National Cancer Institute. ADOPT core measures data files. Updated April 1, 2022. Accessed January 28, 2025. https://www.gis.cancer.gov/research/files.html#soc-dep

[24] Robert Graham Center. Social deprivation index (SDI). Accessed January 28, 2025. https://www.graham-center.org/maps-data-tools/social-deprivation-index.html

[25] Dyer Z. Structural Racism Effect Index. Accessed January 28, 2025. https://www.sreindex.com/

[26] Agency for Toxic Substances and Disease Registry. SVI data & documentation download 2018. Accessed January 28, 2025. https://www.atsdr.cdc.gov/place-health/php/svi/svi-data-documentation-download.html?CDC_AAref_Val=https://www.atsdr.cdc.gov/placeandhealth/svi/data_documentation_download.html

[27] Manson S, Schroeder J, Van Riper D, . IPUMS National historical geographic information system: version 19.0. Published 2024. Accessed February 26, 2026. https://www.ipums.org/projects/ipums-nhgis/d050.V19.0

[28] Centers for Disease Control and Prevention. PLACES: local data for better health. Accessed January 28, 2025. https://www.cdc.gov/places/index.html

[29] Kaalund K, Thoumi A, Bhavsar NA, Labrador A, Cholera R. Assessment of population-level disadvantage indices to inform equitable health policy. Milbank Q. 2022;100(4):1028-1075. doi:10.1111/1468-0009.12588 36454129 PMC9836250

[30] US Census Bureau. TIGER/line shapefiles. Updated December 23, 2025. Accessed February 25, 2026. https://www.census.gov/geographies/mapping-files/time-series/geo/tiger-line-file.html

[31] Kim JH. Multicollinearity and misleading statistical results. Korean J Anesthesiol. 2019;72(6):558-569. doi:10.4097/kja.19087 31304696 PMC6900425

[32] Lou S, Giorgi S, Liu T, Eichstaedt JC, Curtis B. Measuring disadvantage: a systematic comparison of United States small-area disadvantage indices. Health Place. 2023;80:102997. doi:10.1016/j.healthplace.2023.102997 36867991 PMC10038931

[33] Tipirneni R, Schmidt H, Lantz PM, Karmakar M. Associations of 4 geographic social vulnerability indices with US COVID-19 incidence and mortality. Am J Public Health. 2022;112(11):1584-1588. doi:10.2105/AJPH.2022.307018 36108250 PMC9558191

[34] Barber LE, Maliniak ML, Nash R, . A comparison of three area-level indices of neighborhood deprivation and socioeconomic status and their applicability to breast cancer mortality. J Urban Health. 2024;101(1):75-79. doi:10.1007/s11524-023-00811-1 38158547 PMC10897108

[35] Herb J, Dunham L, Stitzenberg K. A comparison of area-level socioeconomic status indices in colorectal cancer care. J Surg Res. 2022;280:304-311. doi:10.1016/j.jss.2022.07.036 36030606

[36] Ramgopal S, Kemal S, Attridge MM, Crowe R, Martin-Gill C, Macy ML. Comparison of neighborhood disadvantage indices on emergency medical services interventions and outcomes for pediatric out-of-hospital emergencies. Acad Pediatr. 2025;25(2):102592. doi:10.1016/j.acap.2024.10.004 39396570

[37] Zolotor A, Huang RW, Bhavsar NA, Cholera R. Comparing social disadvantage indices in pediatric populations. Pediatrics. 2024;154(3):e2023064463. doi:10.1542/peds.2023-064463 39143925 PMC11350100

[38] Bucholz EM, McCormick AD, Ronai C. The child opportunity index and other social determinants of health neighborhood indexes: important considerations for choosing the correct index. JAMA Pediatr. 2024;178(12):1370-1372. doi:10.1001/jamapediatrics.2024.3146 39401022 PMC11581656

[39] Park C, Schappe T, Peskoe S, . A comparison of deprivation indices and application to transplant populations. Am J Transplant. 2023;23(3):377-386. doi:10.1016/j.ajt.2022.11.018 36695687 PMC10226151

[40] Rollings KA, Noppert GA, Griggs JJ, Ibrahim AM, Clarke PJ. Comparing deprivation vs vulnerability index performance using Medicare beneficiary surgical outcomes. JAMA Surg. 2024;159(12):1404-1413. doi:10.1001/jamasurg.2024.4195 39356528 PMC11447624

[41] Jacobs MA, Schmidt S, Hall DE. Choosing the right neighborhood deprivation index. JAMA Surg. 2024;159(12):1414. doi:10.1001/jamasurg.2024.4204 39356526

[42] Srivastava T, Schmidt H, Sadecki E, Kornides ML. Disadvantage indices deployed to promote equitable allocation of COVID-19 vaccines in the US: a scoping review of differences and similarities in design. JAMA Health Forum. 2022;3(1):e214501. doi:10.1001/jamahealthforum.2021.4501 35977227 PMC8903102

[43] Freedman AA, Keenan-Devlin LS, Smart BP, . A multidimensional tool for quantifying structural racism: Application to adverse pregnancy outcomes in Chicago, Illinois. Soc Sci Med. 2025;372:118013. doi:10.1016/j.socscimed.2025.118013 40147332 PMC12011194

