# Supplementary material for: Neighborhood Indices, Income, and Cardiovascular-Kidney-Metabolic Syndrome at the Census Tract Level
Source: JAMA Netw Open. 2026 Apr 9;9(4):e266019. doi: 10.1001/jamanetworkopen.2026.6019 (PMC13067008; doi:10.1001/jamanetworkopen.2026.6019)
Supplement: Supplement 1. — eTable 1. Domains, variables, and methodology for each neighborhood index eTable 2. Comparison of selected domains included in seven neighborhood indices eTable 3. Characteristics of US census tracts excluded in analysis eFigure 1. Geographic heterogeneity in cardiovascular-kidney-metabolic conditions at the census tract-level eFigure 2. Quartile agreement between neighborhood indices and median household income at the census tract-level eFigure 3. Distribution of standardized neighborhood index scores and median household income at the census tract-level across the US eTable 4. Associations between neighborhood indices, median household income, and cardiovascular-kidney-metabolic risk factors at the census tract-level eTable 5. Change in R2 values with addition of neighborhood indices to median household income for cardiovascular-kidney-metabolic risk factors at the census tract-level eTable 6. Unadjusted associations between neighborhood indices, median household income, and cardiovascular-kidney-metabolic conditions at the census tract-level eTable 7. Associations between SREI subdomains and cardiovascular-kidney-metabolic conditions at the census tract-level eTable 8. Change in R2 values for Structural Racism Effect Index subdomains across cardiovascular-kidney-metabolic conditions at the census tract-level eReferences [file jamanetwopen-e266019-s001.pdf]

Supplemental Online Content

Krishnan V, Huang X, MCGowan C, et al. Neighborhood Indices, income, and cardiovascular-kidney-metabolic syndrome at the census tract level. *JAMA Netw. Open*. 2026;9(4):e266019. doi:10.1001/jamanetworkopen.2026.6019

eTable 1. Domains, variables, and methodology for each neighborhood index

eTable 2. Comparison of selected domains included in seven neighborhood indices

eTable 3. Characteristics of US census tracts excluded in analysis

eFigure 1. Geographic heterogeneity in cardiovascular-kidney-metabolic conditions at the census tract-level

eFigure 2. Quartile agreement between neighborhood indices and median household income at the census tract-level

eFigure 3. Distribution of standardized neighborhood index scores and median household income at the census tract-level across the US

eTable 4. Associations between neighborhood indices, median household income, and cardiovascular-kidney-metabolic risk factors at the census tract-level

eTable 5. Change in R2 values with addition of neighborhood indices to median household income for cardiovascular-kidney-metabolic risk factors at the census tract-level

eTable 6. Unadjusted associations between neighborhood indices, median household income, and cardiovascular-kidney-metabolic conditions at the census tract-level

eTable 7. Associations between SREI subdomains and cardiovascular-kidney-metabolic conditions at the census tract-level

eTable 8. Change in R2 values for Structural Racism Effect Index subdomains across cardiovascular-kidney-metabolic conditions at the census tract-level

eReferences

This supplemental material has been provided by the authors to give readers additional information about their work.

© 2026 Krishnan V et al. *JAMA Network Open*.

eTable 1: Domains, variables, and methodology for each neighborhood index

Index	Version	Purpose	Domains	Component Variables	Scoring	Data Sources and Years
Area Deprivation Index (ADI)[1-3]	2015	Measure area-level socioeconomic deprivation to inform health policy and program delivery	Education, income/employment, housing, household characteristics	1. Population age ≥25 years with <9 years of education<br>2. Population age ≥25 years with ≥12 years of education<br>3. Employed population age ≥16 years in white collar occupations<br>4. Median family income<br>5. Income disparity<br>6. Median home value<br>7. Median gross rent<br>8. Median monthly mortgage<br>9. Owner-occupied housing units<br>10. Unemployed civilian labor force ≥16 years of age<br>11. Families below federal poverty line<br>12. Population below 150% of federal poverty line<br>13. Single parent households with children <18 years of age<br>14. Occupied housing units without a motor vehicle<br>15. Occupied housing units without a telephone<br>16. Occupied housing units without complete plumbing<br>17. Occupied housing units with more than one person per room	Ranked scores from 1 to 100 representing national percentile rankings, where higher values reflect higher neighborhood deprivation.<br><br>State rankings also available.	ACS<br><br>2011-2015

© 2026 Krishnan V et al. *JAMA Network Open*.

Index	Version	Purpose	Domains	Component Variables	Scoring	Data Sources and Years
Child Opportunity Index 3.0 (COI)[4,5]	2019	Measure the quality of neighborhood resources and conditions available to support the healthy development of children	Early childhood education (ECE), elementary education, secondary/postsecondary education, educational resources, healthy environments, pollution, safety-related resources, health resources, employment, economic opportunities, concentrated socioeconomic inequity, housing resources, social resources, wealth	1. Public pre-K enrollment<br>2. Private pre-K enrollment<br>3. Reading and math test scores<br>4. Reading and math test score growth<br>5. Poverty-adjusted reading and math test scores<br>6. Advanced Placement course enrollment<br>7. High school graduation rate<br>8. College enrollment in nearby institutions<br>9. School poverty<br>10. Teacher experience<br>11. Adult educational attainment<br>12. Child enrichment-related non-profits<br>13. Walkability<br>14. NatureScore<br>15. Extreme heat exposure<br>16. Fast food restaurant density<br>17. Healthy food retailer density<br>18. Airborne microparticles<br>19. Ozone concentration<br>20. Industrial pollutants in air, water, or soil<br>21. Hazardous waste dump sites<br>22. Vacant housing	Ranked scores from 1 to 100 representing nationally-normed percentile rankings, where higher values reflect greater opportunity.<br><br>State-normed and metro-normed ranking also available, and COI z-scores are available to re-calculate rankings.	ACS, CRDC,<br>ED*Facts*, EPA, NASA, NatureScore, NCCS, NCES CCD,<br>SEDA, and independently collected data.<br><br>2015-2019

© 2026 Krishnan V et al. *JAMA Network Open*.

Index	Version	Purpose	Domains	Component Variables	Scoring	Data Sources and Years
				23. Community safety-related non-profits<br>24. Health insurance coverage<br>25. Health-related non-profits<br>26. Employment rate<br>27. High-skill employment rate<br>28. Full-time year-round earnings<br>29. Poverty rate<br>30. Public assistance rate<br>31. Median household income<br>32. Adults with masters, professional, or doctoral degrees<br>33. Very high-income households<br>34. Adults without a high school degree<br>35. Very low-income households<br>36. Crowded housing<br>37. Broadband access<br>38. Single-parent families<br>39. Non-profit organizations<br>40. Mobility-enhancing friendship networks<br>41. Homeownership rate<br>42. Aggregate home value per capita<br>43. Aggregate capital income per capita<br>44. Aggregate real estate taxes per capita		

© 2026 Krishnan V et al. *JAMA Network Open*.

Index	Version	Purpose	Domains	Component Variables	Scoring	Data Sources and Years
Environmental Justice Index (EJI)[6,7]	2022	Rank the impact of environmental injustice on health to inform public health and policy	Social vulnerability module: R/E minority status, SE status, household characteristics, housing type<br><br>Environmental burden module: air pollution, potentially hazardous & toxic sites, built environment, transportation infrastructure, water pollution<br><br>Health vulnerability: pre-existing chronic disease burden	1. Minority status<br>2. Poverty<br>3. Individuals without HS diploma<br>4. Unemployment<br>5. Housing tenure<br>6. Housing burdened lower-income households<br>7. Lack of health insurance<br>8. Lack of broadband access<br>9. Households with individuals age ≥65 years<br>10. Households with individuals age ≤17 years<br>11. Households with a civilian with a disability<br>12. Households who speak English "less than well"<br>13. Group quarters<br>14. Mobile homes<br>15. Ozone<br>16. PM2.5<br>17. Diesel particulate matter<br>18. Air toxics cancer risk<br>19. National priority list sites for potentially hazardous/toxic sites<br>20. Toxic release inventory sites<br>21. Treatment, storage, and disposal sites	Ranked scores from 0 to 1 representing national percentile rankings, where higher values reflect higher neighborhood deprivation.	ACS, EPA, MDRS, CDC PLACES, and additional sources<br><br>2014-2021

© 2026 Krishnan V et al. *JAMA Network Open*.

Index	Version	Purpose	Domains	Component Variables	Scoring	Data Sources and Years
				22. Risk management plan sites<br>23. Coal mines<br>24. Lead mines<br>25. Recreational parks<br>26. Houses built pre-1980<br>27. Walkability<br>28. High-volume roads<br>29. Railways<br>30. Airports<br>31. Impaired surface water/water pollution<br><br>Excluded in our analyses (Health Vulnerability Module)<br>32. High estimated prevalence of asthma*<br>33. High estimated prevalence of cancer*<br>34. High estimated prevalence of high blood pressure*<br>35. High estimated prevalence of diabetes*<br>36. High estimated prevalence of poor mental health*<br><br>*Excluded as recommended by technical documentation		
Neighborhood Deprivation Index (NDI)[8]	2013-2017	Multi-dimensional measure of socioeconomic	Wealth and income, education, occupation, and housing conditions	1. Median household income<br>2. Households receiving dividends, interest, or rental income	Raw scores from -3.6 to +2.8, where more positive scores reflect	ACS

© 2026 Krishnan V et al. *JAMA Network Open*.

Index	Version	Purpose	Domains	Component Variables	Scoring	Data Sources and Years
		status for cancer research		3. Households receiving public assistance<br>4. Median home value<br>5. Employed in management, business, science, or arts occupation<br>6. Households that are female headed with children age <18 years<br>7. HS diploma or higher<br>8. College degree or higher<br>9. Families with income below federal poverty line<br>10. Unemployment<br><br><u>Excluded in this version</u><br>11. Owner-occupied housing units*<br>12. Households without a telephone*<br>13. Households without complete plumbing facilities*<br><br>*Excluded in this derivation of NDI due to low factor loadings	higher neighborhood deprivation.<br><br>NDI quintiles are also available.	2013-2017
Social Deprivation Index (SDI)[9]	2019	Quantify disadvantage for research and policy	Income/poverty, education, employment, housing, and household characteristics	1. Families with income below federal poverty line<br>2. Population age ≥25 years with <12 years of education<br>3. Unemployment for individuals age 16 to 64 years<br>4. Households living in renter-occupied housing units	Ranked scores from 1 to 100 representing national percentile rankings, where higher values reflect higher	ACS<br><br>2015-2019

© 2026 Krishnan V et al. *JAMA Network Open*.

Index	Version	Purpose	Domains	Component Variables	Scoring	Data Sources and Years
				5. Households living in crowded housing units<br>6. Single parent households with dependent children age <18 years<br>7. Households with no vehicle	neighborhood deprivation.<br><br>Raw measures are also available.	
Structural Racism Effect Index (SREI)[10,11]	2023	Quantify neighborhood disparities due to historic discriminatory policies	Built environment, criminal justice, education, employment, housing, income & poverty, social cohesion, transportation, wealth	1. Building vacancy rate<br>2. Mobile homes<br>3. No internet access<br>4. Cancer risk<br>5. Low food access for SNAP recipients<br>6. Pretrial jail rate<br>7. Total jail rate<br>8. Law enforcement personnel per capita<br>9. Bachelor's degree or higher<br>10. HS diploma<br>11. Per pupil spending<br>12. Unemployment<br>13. White-collar occupation<br>14. Retail job availability<br>15. Housing units without telephone<br>16. Housing units without plumbing<br>17. Crowding	Raw scores from -4.3 to +5.0, where more positive scores reflect higher neighborhood deprivation.<br><br>National percentile ranking also available.	ACS, EPA, USDA, FBI, and additional data sources<br><br>2010-2022

© 2026 Krishnan V et al. *JAMA Network Open*.

Index	Version	Purpose	Domains	Component Variables	Scoring	Data Sources and Years
				18. Group quarters<br>19. Foreclosure risk<br>20. Eviction rate<br>21. Below 100% of federal poverty line<br>22. Below 200% of federal poverty line<br>23. Public assistance<br>24. Family income<br>25. Per capita income<br>26. Supplemental poverty measure<br>27. Changed address in last year<br>28. Single parent households<br>29. Income gap<br>30. Residential segregation<br>31. Carpooled to work<br>32. No motor vehicle access<br>33. Took public transit to work<br>34. Biked to work<br>35. Walked to work<br>36. Transportation cost burden, median income family<br>37. Aggregate home value<br>38. Median real estate taxes paid<br>39. Median home value		

© 2026 Krishnan V et al. *JAMA Network Open*.

Index	Version	Purpose	Domains	Component Variables	Scoring	Data Sources and Years
				40. Median gross rent<br>41. Median monthly mortgage<br>42. Owner-occupied homes		
Social Vulnerability Index (SVI)[12,13]	2018	For hazardous events (weather, chemical spill, etc.) preparation and response, and emergency resource allocation	SE status, household composition and disability, minority status and language, housing type and transportation	1. Below poverty line<br>2. Unemployment<br>3. Income<br>4. No HS diploma<br>5. Households with individuals age ≥65 years<br>6. Households with individuals age ≤17 years<br>7. Households with a civilian with a disability<br>8. Single parent households<br>9. Minority status<br>10. Households who speak English "less than well"<br>11. Multi-unit structures for housing<br>12. Mobile home<br>13. Households with crowding<br>14. Households without vehicles<br>15. Group quarters	Ranked scores from 0 to 1 representing national percentile rankings, where higher values reflect higher neighborhood deprivation.	ACS<br><br>2014-2018

Abbreviations include high school (HS), American Community Survey (ACS), U.S. Department of Education Office for Civil Rights Data Collection (CRDC), Environmental Protection Agency (EPA), Federal Bureau of Investigation (FBI), National Aeronautics and Space Administration (NASA),

© 2026 Krishnan V et al. *JAMA Network Open*.

National Center for Charitable Statistics (NCCS), National Center for Education Statistics Common Core of Data (NCES CCD), Stanford Education Data Archive (SEDA), US Department of Agriculture (USDA), US Mine Safety and Health Administration Mine Data Retrieval System (MDRS). EDFacts data from the US Department of Education.

© 2026 Krishnan V et al. *JAMA Network Open*.

eTable 2: Comparison of selected domains included in seven neighborhood indices

	Total number of component variables	Education, Employment, & Poverty	Housing & Household Characteristics	Built Environment & Community Resources	Natural Environment & Hazards	Criminal Justice & Safety	Minority Status	Health Insurance
**ADI**	17	X	X					
**COI**	44	X	X	X	X	X		X
**EJI**	36*	X	X	X	X		X	X
**NDI**	13*	X	X					
**SDI**	7	X	X					
**SREI**	42	X	X	X	X	X		
**SVI**	15	X	X				X	

(*) Indicates total number of variables, including those excluded in this analysis. See eTable 1 for additional details.

Abbreviations include Area Deprivation Index [ADI], Child Opportunity Index [COI], Environmental Justice Index [EJI], Neighborhood Deprivation Index [NDI], Social Deprivation Index [SDI], Structural Racism Effect Index [SREI], Social Vulnerability Index [SVI].

© 2026 Krishnan V et al. *JAMA Network Open*.

eTable 3: Characteristics of US census tracts excluded in analysis

	Number of census tracts with missing variable	Median (IQR)
**Total Population**	317	3664 (2465.5, 5088)
**Median Age (years)**	317	39.9 (33.5, 45.8)
**Median Household Income (dollars)**	317	71050 (45018.5, 106250)
**Index Scores**		
ADI	325	26.86 (11.84, 53.02)
COI	37	62.00 (26.75, 88.00)
EJI	660	0.49 (0.22, 0.78)
NDI	4513	-0.49 (-1.22, 0.19)
SDI	37	43.0 (16.0, 78.0)
SREI	422	-0.48 (-1.21, 0.46)
SVI	197	0.38 (0.15, 0.71)
**CKM Conditions Prevalence (%)**		
Coronary Heart Disease	1999	5.2 (3.7, 7.1)
Stroke	1999	2.9 (2.1, 4.0)
Chronic Kidney Disease	1999	2.7 (2.1, 3.5)
Obesity	1999	28.7 (23.4, 35.1)
Hypertension	1999	29.9 (24.5, 35.8)
Diabetes	1999	9.3 (7.0, 12.7)

Values represent median (interquartile range [IQR]). Among the 72,337 census tracts available in CDC PLACES, 6,861 were missing data for at least one exposure, covariate, or outcome. Index scores represent crude, unstandardized values. Data on total population, median age, and median household income were obtained from the 2015-2019 American Community Survey. Prevalence data for cardiovascular-kidney-metabolic conditions were obtained from CDC PLACES.

Abbreviations include Area Deprivation Index [ADI], Child Opportunity Index [COI], Environmental Justice Index [EJI], Neighborhood Deprivation Index [NDI], Social Deprivation Index [SDI], Structural Racism Effect Index [SREI], Social Vulnerability Index [SVI].

© 2026 Krishnan V et al. *JAMA Network Open*.

eFigure 1: Geographic heterogeneity in cardiovascular-kidney-metabolic conditions at the census tract-level

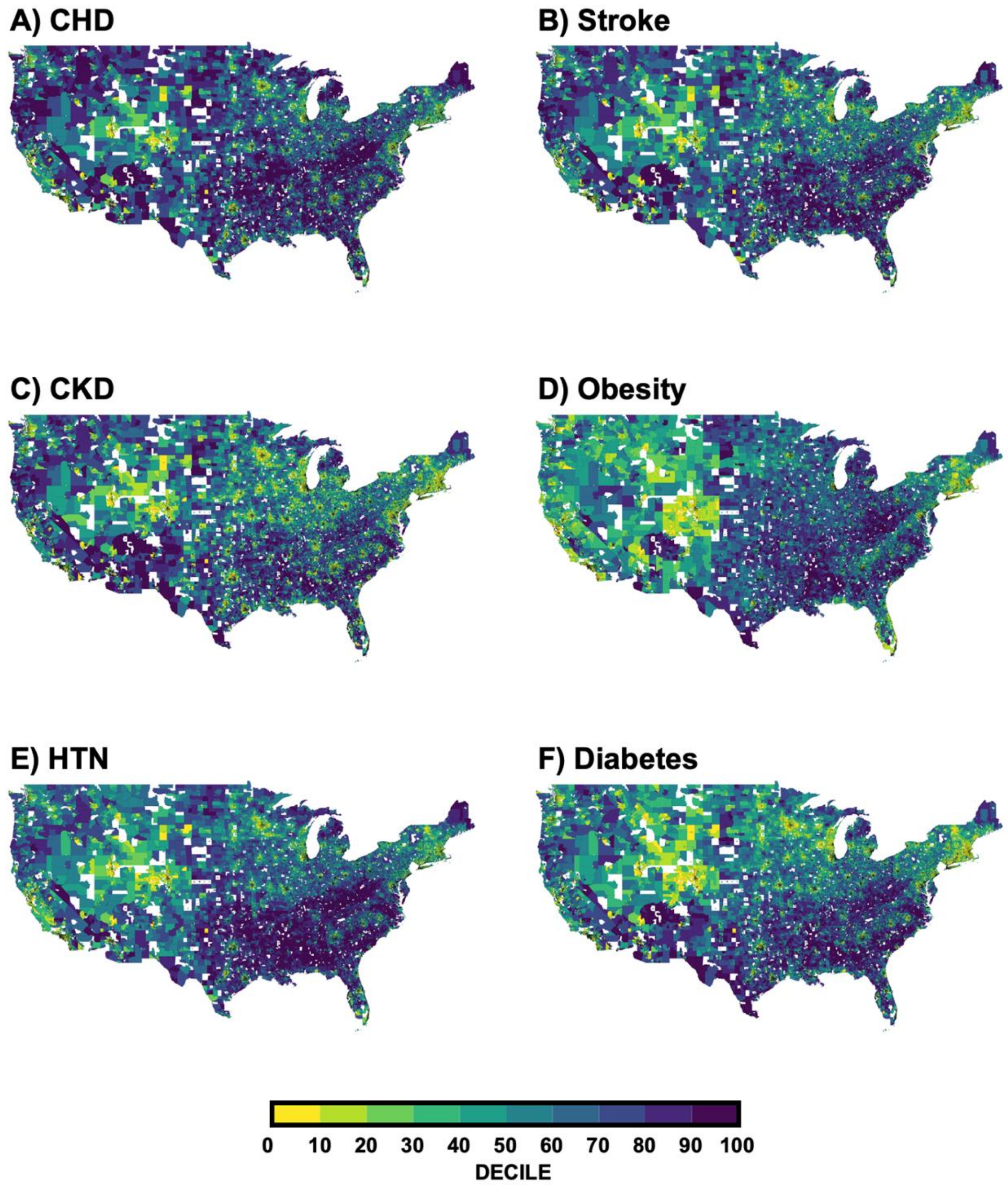

Map of census-tract level data for the prevalence of cardiovascular-kidney-metabolic [CKM] conditions of (A) coronary heart disease [CHD], (B) stroke, (C) chronic kidney disease [CKD], (D) obesity, (E) hypertension [HTN], and (F) diabetes. Color scale represents deciles of crude prevalence data for each condition. Darker shading (i.e., higher deciles) represents greater prevalence.

© 2026 Krishnan V et al. *JAMA Network Open*.

eFigure 2: Quartile agreement between neighborhood indices and median household income at the census tract-level

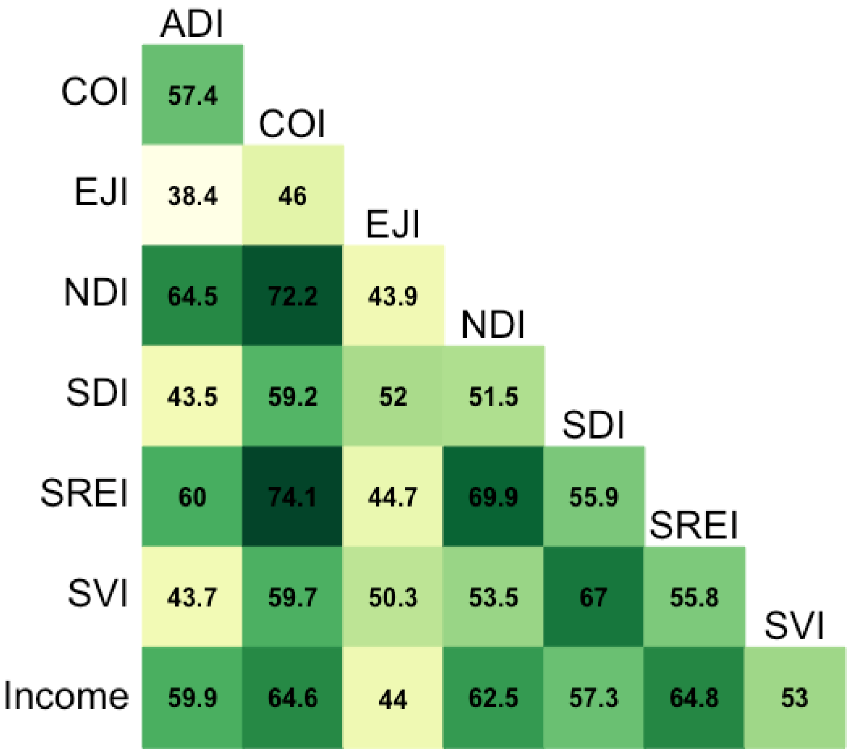

Heatmaps for pairwise analyses across crude index scores and median household income assessing quartile agreement by two neighborhood measures (i.e., proportion of census tracts concordantly categorized as the same quartile, values represent %). Darker shading represents higher values for agreement.

Abbreviations include Area Deprivation Index [ADI], Child Opportunity Index [COI], Environmental Justice Index [EJI], Neighborhood Deprivation Index [NDI], Social Deprivation Index [SDI], Structural Racism Effect Index [SREI], Social Vulnerability Index [SVI].

© 2026 Krishnan V et al. *JAMA Network Open*.

eFigure 3: Distribution of standardized neighborhood index scores and median household income at the census tract-level across the US

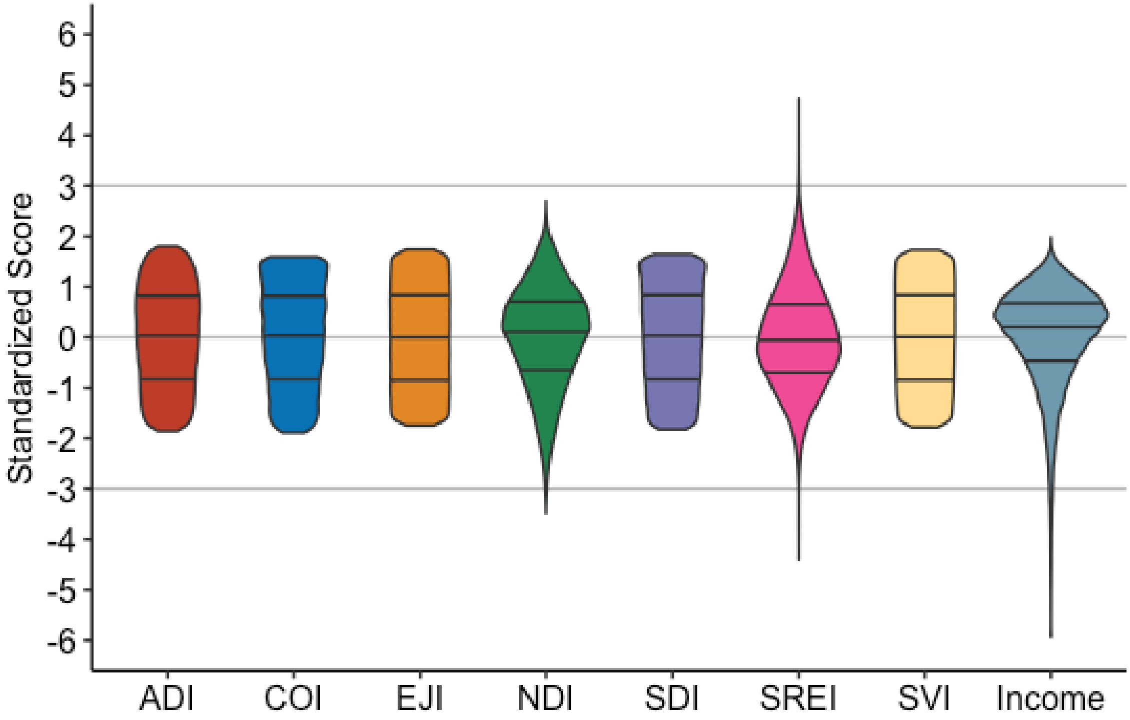

Violin plots represent the distribution of each neighborhood index and median household income after standardization.

Abbreviations include Area Deprivation Index [ADI], Child Opportunity Index [COI], Environmental Justice Index [EJI], Neighborhood Deprivation Index [NDI], Social Deprivation Index [SDI], Structural Racism Effect Index [SREI], Social Vulnerability Index [SVI].

© 2026 Krishnan V et al. *JAMA Network Open*.

eTable 4: Associations between neighborhood indices, median household income, and cardiovascular-kidney-metabolic risk factors at the census tract-level

	Obesity		Hypertension		Diabetes	
	Beta (SE)	r^2 (SE)	Beta (SE)	r^2 (SE)	Beta (SE)	r^2 (SE)
**ADI**	0.773 (0.003)	0.629 (0.002)	0.679 (0.003)	0.570 (0.002)	0.648 (0.003)	0.454 (0.003)
**COI**	0.736 (0.003)	0.544 (0.003)	0.705 (0.003)	0.571 (0.002)	0.820 (0.003)	0.639 (0.002)
**EJI**	0.413 (0.004)	0.229 (0.003)	0.474 (0.004)	0.338 (0.003)	0.617 (0.003)	0.389 (0.003)
**NDI**	0.784 (0.003)	0.623 (0.002)	0.681 (0.003)	0.557 (0.002)	0.770 (0.003)	0.590 (0.002)
**SDI**	0.521 (0.004)	0.283 (0.003)	0.595 (0.004)	0.406 (0.003)	0.775 (0.003)	0.503 (0.002)
**SREI**	0.798 (0.003)	0.632 (0.002)	0.777 (0.002)	0.668 (0.002)	0.842 (0.002)	0.679 (0.002)
**SVI**	0.510 (0.004)	0.305 (0.003)	0.581 (0.003)	0.435 (0.003)	0.755 (0.003)	0.549 (0.002)
**Income**	0.624 (0.003)	0.432 (0.003)	0.591 (0.003)	0.462 (0.003)	0.636 (0.003)	0.431 (0.002)

Values represent beta coefficients (standard error [SE]) and r^2 values from multivariable linear regression models between neighborhood measures and cardiovascular-kidney-metabolic [CKM] risk factors, adjusted for census tract population and median age. Bootstrapping with 500 replicates for SE for r^2 values.

All neighborhood measures and CKM risk factors were standardized before linear regression was performed. All neighborhood measures were significantly associated with CKM risk factors ($p<0.05$).

Abbreviations include Area Deprivation Index [ADI], Child Opportunity Index [COI], Environmental Justice Index [EJI], Neighborhood Deprivation Index [NDI], Social Deprivation Index [SDI], Structural Racism Effect Index [SREI], Social Vulnerability Index [SVI].

© 2026 Krishnan V et al. *JAMA Network Open*.

eTable 5: Change in R^2 values with addition of neighborhood indices to median household income for cardiovascular-kidney-metabolic risk factors at the census tract-level

	Obesity	Hypertension	Diabetes
	Δr^2 (SE)	Δr^2 (SE)	Δr^2 (SE)
**ADI**	0.200 (0.002)	0.120 (0.002)	0.066 (0.002)
**COI**	0.116 (0.002)	0.111 (0.002)	0.209 (0.002)
**EJI**	0.006 (0)	0.026 (0.001)	0.084 (0.001)
**NDI**	0.192 (0.002)	0.097 (0.002)	0.159 (0.002)
**SDI**	0 (0)	0.018 (0.001)	0.100 (0.002)
**SREI**	0.201 (0.002)	0.208 (0.002)	0.251 (0.002)
**SVI**	0.008 (0.001)	0.042 (0.001)	0.148 (0.002)

Values represent delta r^2 (standard error [SE]). Delta r^2 values were derived after each place-base index was individually added to multivariable linear regression models between median household income and CKM risk factors, adjusted for census tract population and median age. All variance inflation factors for these models (median household income + index + covariates) were less than 5. Bootstrapping with 500 replicates for SE. Values <0.001 are reported as 0.

All neighborhood measures and CKM risk factors were standardized before linear regression was performed.

Abbreviations include Area Deprivation Index [ADI], Child Opportunity Index [COI], Environmental Justice Index [EJI], Neighborhood Deprivation Index [NDI], Social Deprivation Index [SDI], Structural Racism Effect Index [SREI], Social Vulnerability Index [SVI].

© 2026 Krishnan V et al. *JAMA Network Open*.

eTable 6: Unadjusted associations between neighborhood indices, median household income, and cardiovascular-kidney-metabolic conditions at the census tract-level

	CKM Outcomes						CKM Risk Factors					
	Coronary Heart Disease		Stroke		Chronic Kidney Disease		Obesity		Hypertension		Diabetes	
	Beta (SE)	r^2	Beta (SE)	r^2	Beta (SE)	r^2	Beta (SE)	r^2	Beta (SE)	r^2	Beta (SE)	r^2
**ADI**	0.623 (0.003)	0.388	0.652 (0.003)	0.426	0.626 (0.003)	0.392	0.791 (0.002)	0.626	0.626 (0.003)	0.392	0.647 (0.003)	0.419
**COI**	0.542 (0.003)	0.294	0.667 (0.003)	0.445	0.695 (0.003)	0.483	0.733 (0.003)	0.537	0.557 (0.003)	0.310	0.740 (0.003)	0.547
**EJI**	0.287 (0.004)	0.082	0.456 (0.003)	0.208	0.495 (0.003)	0.245	0.450 (0.003)	0.203	0.319 (0.004)	0.101	0.538 (0.003)	0.290
**NDI**	0.559 (0.003)	0.312	0.654 (0.003)	0.428	0.665 (0.003)	0.442	0.785 (0.002)	0.616	0.560 (0.003)	0.314	0.716 (0.003)	0.513
**SDI**	0.353 (0.004)	0.125	0.522 (0.003)	0.272	0.572 (0.003)	0.327	0.512 (0.003)	0.262	0.335 (0.004)	0.112	0.592 (0.003)	0.351
**SREI**	0.579 (0.003)	0.335	0.722 (0.003)	0.521	0.725 (0.003)	0.526	0.791 (0.002)	0.626	0.632 (0.003)	0.399	0.769 (0.002)	0.592
**SVI**	0.413 (0.004)	0.171	0.567 (0.003)	0.321	0.625 (0.003)	0.391	0.524 (0.003)	0.275	0.398 (0.004)	0.158	0.645 (0.003)	0.416
**Income**	0.558 (0.003)	0.311	0.618 (0.003)	0.382	0.630 (0.003)	0.397	0.653 (0.003)	0.426	0.518 (0.003)	0.268	0.621 (0.003)	0.385

Values represent beta coefficients (standard error [SE]) and r^2 values from multivariable linear regression models between neighborhood measures and cardiovascular-kidney-metabolic [CKM] conditions, unadjusted for covariates.

All neighborhood measures and CKM conditions were standardized before linear regression was performed. All neighborhood measures were significantly associated with CKM conditions ($p<0.05$).

Abbreviations include Area Deprivation Index [ADI], Child Opportunity Index [COI], Environmental Justice Index [EJI], Neighborhood Deprivation Index [NDI], Social Deprivation Index [SDI], Structural Racism Effect Index [SREI], Social Vulnerability Index [SVI].

© 2026 Krishnan V et al. *JAMA Network Open*.

eTable 7: Associations between SREI subdomains and cardiovascular-kidney-metabolic conditions at the census tract-level

	CKM Outcomes						CKM Risk Factors					
	**Coronary Heart Disease**		**Stroke**		**Chronic Kidney Disease**		**Obesity**		**Hypertension**		**Diabetes**	
	Beta (SE)	r^2	Beta (SE)	r^2	Beta (SE)	r^2	Beta (SE)	r^2	Beta (SE)	r^2	Beta (SE)	r^2
**Built Environment**	0.577 (0.003)	0.519	0.651 (0.003)	0.512	0.629 (0.003)	0.472	0.570 (0.003)	0.391	0.628 (0.003)	0.520	0.671 (0.003)	0.493
**Criminal Justice**	0.253 (0.003)	0.273	0.286 (0.004)	0.199	0.249 (0.004)	0.167	0.233 (0.004)	0.143	0.346 (0.003)	0.272	0.294 (0.004)	0.161
**Education**	0.570 (0.003)	0.513	0.564 (0.003)	0.416	0.600 (0.003)	0.441	0.628 (0.003)	0.457	0.536 (0.003)	0.422	0.649 (0.003)	0.468
**Employment**	0.570 (0.003)	0.516	0.627 (0.003)	0.489	0.619 (0.003)	0.467	0.665 (0.003)	0.506	0.589 (0.003)	0.481	0.632 (0.003)	0.452
**Housing**	0.379 (0.004)	0.329	0.485 (0.004)	0.313	0.505 (0.004)	0.316	0.371 (0.004)	0.203	0.371 (0.004)	0.268	0.479 (0.004)	0.265
**Income and Poverty**	0.716 (0.003)	0.600	0.806 (0.003)	0.613	0.857 (0.003)	0.664	0.668 (0.003)	0.429	0.672 (0.003)	0.497	0.845 (0.003)	0.618
**Social Cohesion**	0.496 (0.004)	0.377	0.752 (0.003)	0.504	0.756 (0.003)	0.494	0.585 (0.004)	0.322	0.635 (0.004)	0.428	0.750 (0.003)	0.458
**Transportation**	0.438 (0.003)	0.398	0.400 (0.003)	0.276	0.382 (0.003)	0.249	0.555 (0.003)	0.392	0.467 (0.003)	0.368	0.426 (0.003)	0.253
**Wealth**	0.619 (0.003)	0.541	0.581 (0.003)	0.411	0.556 (0.003)	0.373	0.713 (0.003)	0.529	0.601 (0.003)	0.467	0.552 (0.003)	0.338

Values represent beta coefficients (standard error [SE]) and r^2 from multivariable linear regression models between Structural Racism Effect Index [SREI] subdomains and cardiovascular-kidney-metabolic [CKM] conditions, adjusted for census tract population and median age.

All SREI subdomains and CKM conditions were standardized before linear regression was performed. All SREI subdomains were significantly associated with CKM conditions ($p<0.05$).

© 2026 Krishnan V et al. *JAMA Network Open*.

eTable 8: Change in R^2 values for Structural Racism Effect Index subdomains across cardiovascular-kidney-metabolic conditions at the census tract-level

	CKM Outcomes			CKM Risk Factors		
	**Coronary Heart Disease**	**Stroke**	**Chronic Kidney Disease**	**Obesity**	**Hypertension**	**Diabetes**
	Δr^2 (SE)	Δr^2 (SE)	Δr^2 (SE)	Δr^2 (SE)	Δr^2 (SE)	Δr^2 (SE)
**Built Environment**	0.029 (0.001)	0.047 (0.001)	0.026 (0.001)	0.021 (0.001)	0.069 (0.001)	0.044 (0.001)
**Criminal Justice**	0.007 (0)	0.012 (0.001)	0.005 (0)	0.003 (0)	0.036 (0.001)	0.015 (0.001)
**Social Cohesion**	0 (0)	0.038 (0.001)	0.029 (0.001)	0.022 (0.001)	0.036 (0.001)	0.039 (0.001)
**Transportation**	0.011 (0.001)	0.007 (0.001)	0.005 (0)	0.020 (0.001)	0.021 (0.001)	0.009 (0.001)
**Base Model**	0.667 (0.002)	0.655 (0.002)	0.690 (0.002)	0.634 (0.002)	0.584 (0.002)	0.662 (0.002)

Values represent delta r^2 (standard error [SE]) after each additional Structural Racism Effect Index [SREI] subdomain was individually added to a base multivariable linear regression model for CKM conditions, adjusted for census tract population and median age. All variance inflation factors for these models were less than 5. Bootstrapping with 500 replicates for SE. Values <0.001 are reported as 0.

All SREI subdomains and CKM conditions were standardized before linear regression was performed. Base model includes education, employment, housing, income and poverty, and wealth subdomains of SREI and covariates.

© 2026 Krishnan V et al. *JAMA Network Open*.

eREFERENCES

1. University of Wisconsin School of Medicine and Public Health. 2015 Area Deprivation Index v3.1. Accessed Jan 28, 2025. https://www.neighborhoodatlas.medicine.wisc.edu/

2. Kind AJH, Buckingham WR. Making Neighborhood-Disadvantage Metrics Accessible - The Neighborhood Atlas. *N Engl J Med*. Jun 28 2018;378(26):2456-2458. doi:10.1056/NEJMp1802313

3. Kind AJ, Jencks S, Brock J, et al. Neighborhood socioeconomic disadvantage and 30-day rehospitalization: a retrospective cohort study. *Ann Intern Med*. Dec 2 2014;161(11):765-74. doi:10.7326/M13-2946

4. Child Opportunity Index 3.0-2021 Data for 2010 Census Tracts. diversitydatakids.org. Accessed Jan 28, 2025. https://www.diversitydatakids.org/research-library/child-opportunity-index-30-2021-census-tract-data

5. Noelke C, McArdle N, DeVoe B, et al. Child Opportunity Index 3.0 Technical Documentation. diversitydatakids.org, Brandeis University. https://diversitydatakids.org/research-library/coi-30-technical-documentation.

6. Environmental Justice Index. Centers for Disease Control and Prevention and Agency for Toxic Substances Disease Registry. Accessed Jan 28, 2025. https://atsdr.cdc.gov/place-health/php/eji/eji-data-download.html

7. Technical Documentation for the Environmental Justice Index 2022. Accessed Jan 28, 2025. https://www.atsdr.cdc.gov/place-health/media/pdfs/2024/07/EJI-2022-Documentation-508.pdf

8. ADOPT Core Measures Data Files. GIS Portal for Cancer Research, National Cancer Institute. Accessed Jan 28, 2025. https://www.gis.cancer.gov/research/files.html#soc-dep

9. Social deprivation index (SDI), 2015-2019. Robert Graham Center - Policy Studies in Family Medicine & Primary Care. Accessed Jan 28, 2025. https://www.graham-center.org/maps-data-tools/social-deprivation-index.html

10. Structural Racism Effect Index. Accessed Jan 28, 2025. https://www.sreindex.com/

11. Dyer Z, Alcusky MJ, Galea S, Ash A. Measuring The Enduring Imprint Of Structural Racism On American Neighborhoods. *Health Aff (Millwood)*. Oct 2023;42(10):1374-1382. doi:10.1377/hlthaff.2023.00659

12. Social Vulnerability Index 2018 Database United States. Centers for Disease Control and Prevention, Agency for Toxic Substances and Disease Registry Geospatial Research, Analysis, and Services Program. Accessed Jan 28, 2025. https://www.atsdr.cdc.gov/placeandhealth/svi/data_documentation_download.html

13. CDC SVI 2018 Documentation. Accessed Jan 28, 2025. https://svi.cdc.gov/map25/data/docs/SVI2018Documentation_01192022_1.pdf
