# Supplementary material for: Neighborhood Indices, Income, and Cardiovascular-Kidney-Metabolic Syndrome at the Census Tract Level
Source: JAMA Netw Open. 2026 Apr 9;9(4):e266019. doi: 10.1001/jamanetworkopen.2026.6019 (PMC13067008; doi:10.1001/jamanetworkopen.2026.6019)
Supplement: Supplement 2. — Data Sharing Statement [file jamanetwopen-e266019-s002.pdf]

Data Sharing Statement

Data
Data available: Yes
Data types: Data (not involving human participants)
How to access data: publicly available
When available: With publication

Supporting Documents
Document types: None

Additional Information
Who can access the data: publicly available
Types of analyses: publicly available
Mechanisms of data availability: publicly available
Any additional restrictions: publicly available
